# Supplementary material for: IFT139 regulates Hedgehog signaling and cilia structure through ciliary protein localization
Source: Biol Open. 2025 Oct 16;14(10):bio062040. doi: 10.1242/bio.062040 (PMC12570150; doi:10.1242/bio.062040)
Supplement: Supplementary information [file biolopen-14-062040-s1.pdf]

S1A

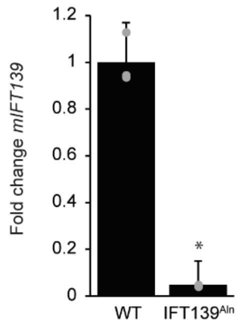

S1B

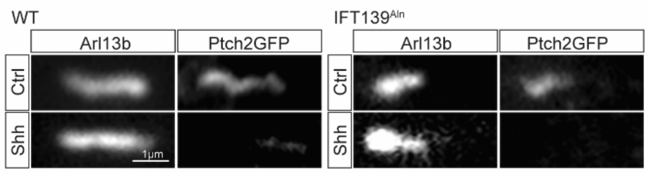

S1C

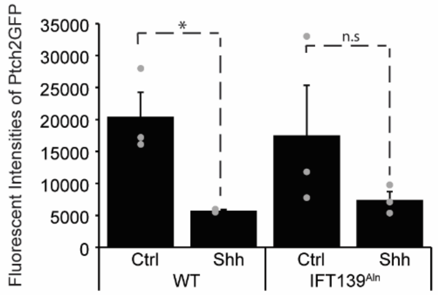

S1D

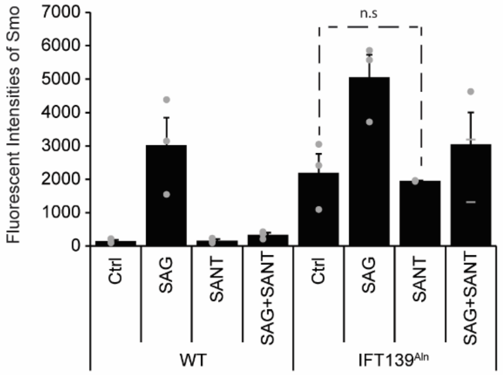

S1E

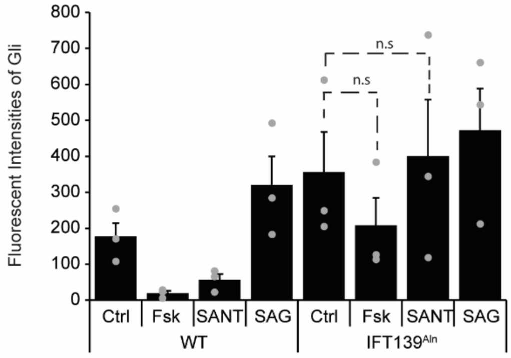

S1F

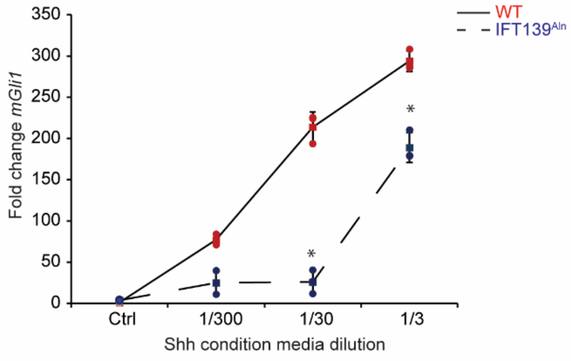

Biology Open • Supplementary information

Fig. S1. Supplementary to Fig. 1.
(A). m*IFT139* expression levels were measured in Wild-type (WT) or IFT139^{Aln} MEFs by qRT-PCR. Bars show average fold-change for three replicates (grey dots) and error bars show SD. Data was normalized to WT. * denotes statistical significance, p<0.05 by paired t-test.
(B) Immunofluorescence images of primary cilia in wild-type (WT) or IFT139 ^{Aln} cells stably expressing Ptch2GFP, incubated with control (ctrl) media or Shh condition media (Shh). Cilia were detected by staining for endogenous Arl13B. Scale bar = 1µm.
(C) Quantification of the experiment in (B). Bars represent average ciliary intensity, and error bars show SD. 100 cilia were measured per condition per experiment for 3 independent experiments (grey dots). * denotes statistical significance p<0.05, n.s denotes not statistically significant, p>0.05 by one-way ANOVA.

(D) Quantification of ciliary levels of endogenous Smo following treatment with control media (ctrl), SAG (1µM), and/or SANT (10 µM). Cilia were detected by staining for endogenous Arl13B. Bars represent average ciliary intensity and error bars show. 100 cilia were measured per condition per experiment for 3 independent experiments (grey dots). n.s denotes not statistically significant P>0.05 by one-way ANOVA.
(E). Quantification of ciliary levels of endogenous Gli following treatment with control media (ctrl), SAG (1µM), SANT (10 µM) or Forskolin (Fsk) (10 µM). Cilia were detected by staining for endogenous Arl13B. Bars represent average ciliary intensity and error bars show SD. 100 cilia were measured per condition per experiment for 3 independent experiments (grey dots). n.s denotes not statistically significant P>0.05 by one-way ANOVA.

(F) Wild-type (WT)(red) or IFT139^{Aln} (blue) MEFs were incubated with control (ctrl) media or Shh condition media (Shh) at different dilutions for 24 hours, and Hh signaling was measured by qRT-PCR for *mGli1*. Lines show average (colored square) fold-change for three replicates (colored dots), and error bars show SD. Data was normalized to ctrl. * denotes statistical significance, p<0.05 by one-way ANOVA.

Biology Open • Supplementary information

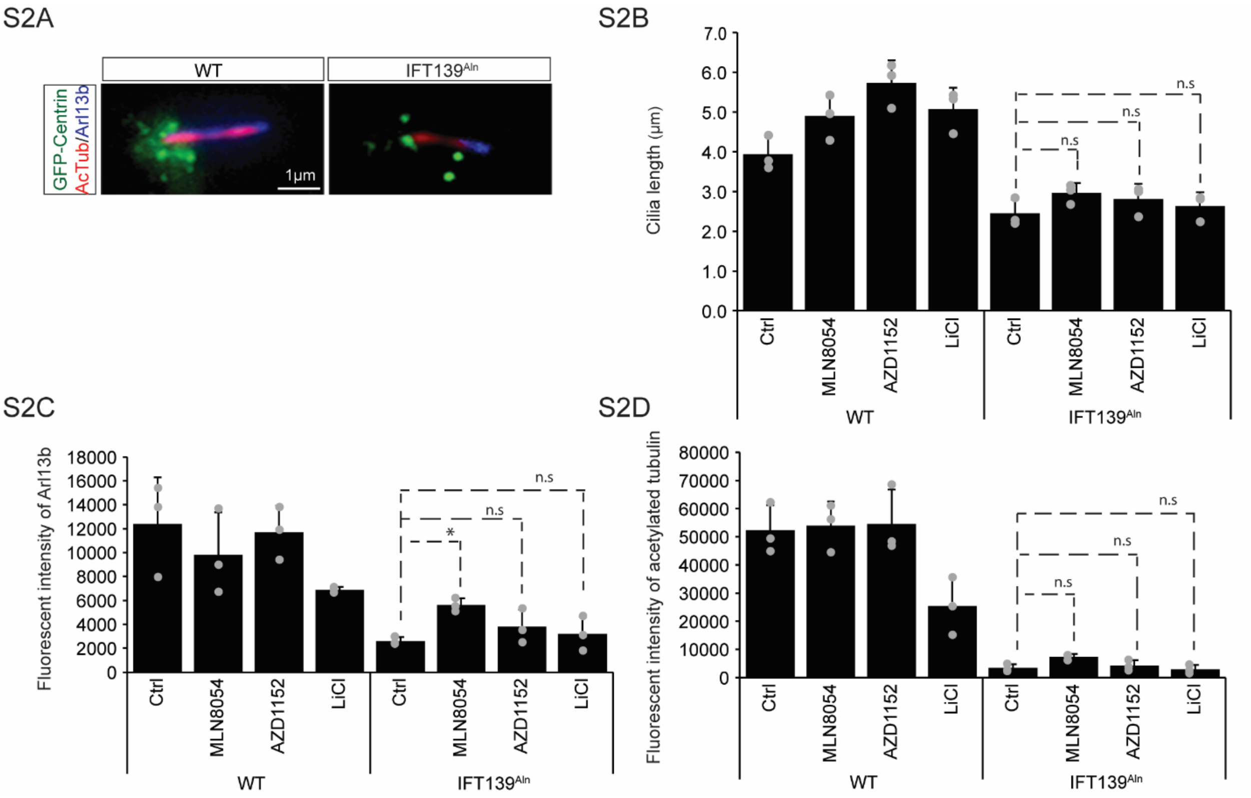

Fig. S2. Supplementary to Fig. 2.

(A). Immunofluorescence images of the primary cilium in wild-type (WT) or IFT139^{Aln} cells expressing CentrinGFP. Cilia were stained for Arl13b (blue) and Acetylated tubulin (red). Scale bar = 1μm.
(B). Quantification of cilia length in wild-type (WT) or IFT139^{Aln} MEF cells treated with control (ctrl, DMSO), aurora kinase A inhibitor (MLN8054, 5nM), aurora kinase B inhibitor (AZD1152, 5nM), or LiCl (5mM). Bars represent average cilia length (Arl13b staining, in μm), and error bars show SD. 150 cilia were measured per condition per experiment for 3 independent experiments (grey dots). n.s denotes not statistically significant p>0.05 by one-way ANOVA.
(C). Quantification of ciliary intensity of endogenous Arl13b in wild-type (WT) or IFT139^{Aln} MEF cells treated with control (ctrl, DMSO), aurora kinase A inhibitor (MLN8054, 5nM), aurora kinase B inhibitor (AZD1152, 5nM), or LiCl (5mM). Bars represent average ciliary intensity, and error bars show SD. 100 cilia were measured per condition per experiment for 3 independent experiments (grey dots). * denotes statistical significance, p<0.05. n.s denotes not statistically significant p>0.05 by one-way ANOVA.
(D) Quantification of ciliary intensity of endogenous acetylated tubulin in wild-type (WT) or IFT139 Aln MEF cells treated with control (ctrl, DMSO), aurora kinase A inhibitor (MLN8054, 5nM), aurora kinase B inhibitor (AZD1152, 5nM), or LiCl (5mM). Bars represent average ciliary intensity, and error bars show SD. 100 cilia were measured per condition per experiment for 3 independent experiments (grey dots). n.s denotes not statistically significant p>0.05 by one-way ANOVA.

S3A

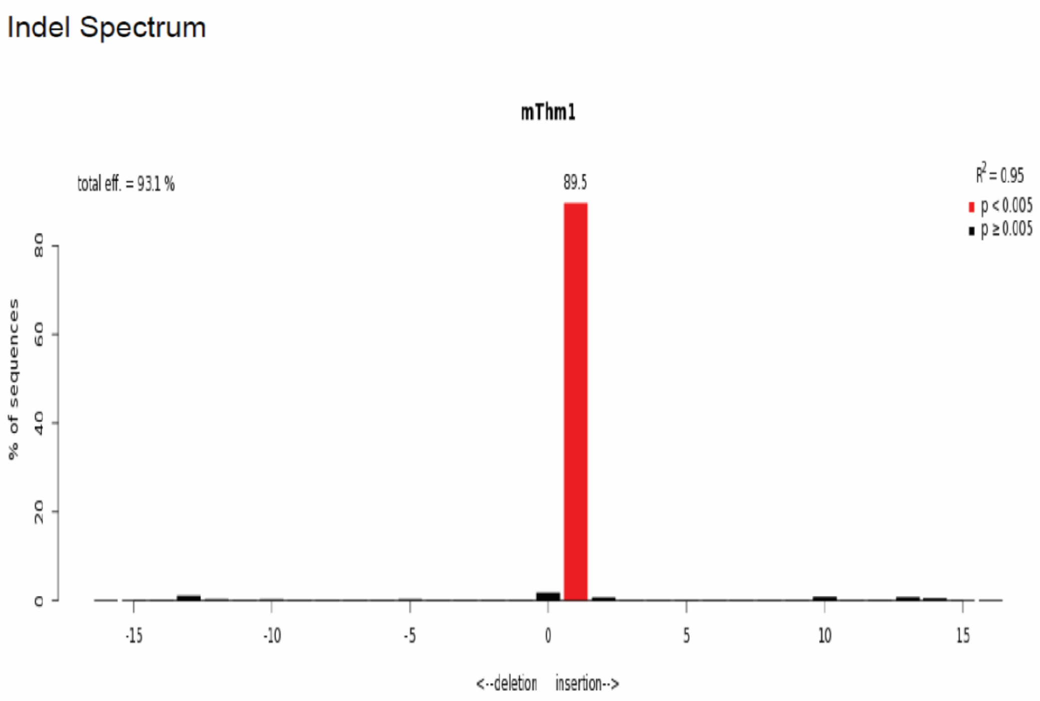

S3B

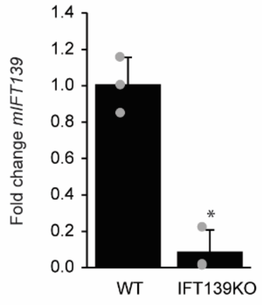

S3C

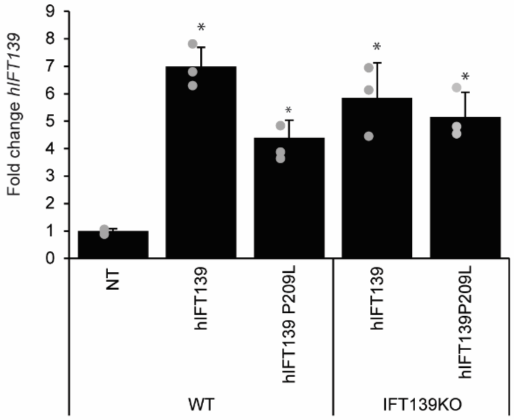

S3D

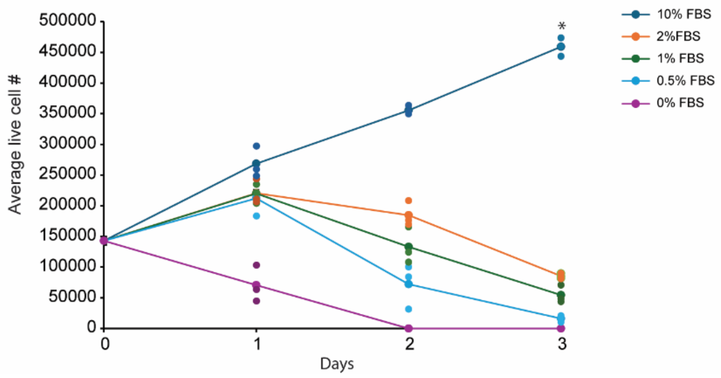

Biology Open • Supplementary information

Fig. S3. Supplementary to Fig. 3.

(A) Indel spectrum from TIDE analysis for C3H10T1/2 IFT139 knockout cells (KO). Red bar indicates insertion at endogenous locus in 89.5% of the sequences at P<0.005, with a predicated editing efficiency of 93.1%.

(B) m*IFT139* expression levels were measured in wild-type (WT) or IFT139KO C3H10T1/2 by qRT-PCR. Bars show average fold-change for three replicates (grey dots), and error bars show SD. Data was normalized to WT. * denotes statistical significance, p<0.05 by paired t-test.

(C) h*IFT139* expression levels were measured in wild-type (WT) or IFT139KO C3H10T1/2 cells expressing wild-type hIFT139 or hIFT139P209L by qRT-PCR. Bars show average fold-change for three replicates (grey dots), and error bars show SD. Data were normalized to WT NT (non-treated control). * denotes statistical significance, p<0.05 as compared to WT NT by one-way ANOVA.

(D) Cell proliferation was measured as the average total live cell number. C3H10T1/2 WT cells grew in media with 10% FBS (dark blue), 2% FBS (orange), 1% FBS (green), 0.5% FBS (light blue) and 0% FBS (magenta) and counted for three consecutive days. The lines represent average live cell number with colored dots represent three replicates. * denotes statistical significance, p<0.05 by one-way ANOVA.

Table S1. Plasmid and primer information.

gRNA

**Target Gene**	**sequence**
mIFT139 gRNA (exon2)	ttccgattttaccacgccta

Primers for cloning

**Gene**	**Vector**	**Forward primer**	**Reverse primer**
hIFT139 P209L CCG>CTG	pHAGE-hygro	caaaagcaggaaggaagctcagaaaattcacg attatctgg	ccagataatcgtgaattttctgagcttccttcctgct tttg
hIFT139 Wildtype (WT)	pHAGE-blast	GCCAGAATTCGGCGCGCCAA TGGACTCGCAGGAATTGAA	GAATTCGCGGCCGCTAGTCAA GGTCTTAAAGACGCAC
mIFT139 gRNA	Px459	Cacc G TTCCGATTTTACCACGCCTA	AAACTAGGCGTGGTAAAATC GGAAC

Sequencing Primers

**Primer name**	**primer sequence**
U6 (for Px459)	GACTATCATATGCTTACCGT
CMV (for pHAGE)	CGCAAATGGGCGGTAGGCGTG

QPCR primers

**Gene**	**Forward primer**	**Reverse primer**
mCyclo (cyclophilin A)	GGAGATGGCACAGGAGGAA	GCCCGTAGTGCTTCAGCTT
mGli1	TACCATGAGCCCTTCTTTAGGA	GCATCATTGAACCCCGAGTAG
mIFT139 (Ttc21b)	GTTTTTATGGCACATTGGTCGTC	TCGGCCCCTCATTGCTATCA
hCyclo (cyclophilin A)	GGCAAATGCTGGACCCAACACA	TGCTGGTCTTGCCATTCCTGGA
hIFT139 (Ttc21b)	ACCAGACAGTTGAGACAGCAC	TGGAAGCCTTCTCTATATCCCC

TIDE sequencing primers

**Gene**	**Forward primer**	**Reverse primer**
mIFT139	AGGGGGAGGAAATTACCCTGA	CGCCACTGTTCTTTAGTTGCC

Biology Open • Supplementary information
